# Artificial intelligence assists precision medicine in cancer treatment

**DOI:** 10.3389/fonc.2022.998222

**Published:** 2023-01-04

**Authors:** Jinzhuang Liao, Xiaoying Li, Yu Gan, Shuangze Han, Pengfei Rong, Wei Wang, Wei Li, Li Zhou

**Affiliations:** ^1^ Department of Radiology, The Third Xiangya Hospital of Central South University, Changsha, Hunan, China; ^2^ Cell Transplantation and Gene Therapy Institute, The Third Xiangya Hospital, Central South University, Changsha, Hunan, China; ^3^ Department of Pathology, The Xiangya Hospital of Central South University, Changsha, Hunan, China

**Keywords:** artificial intelligence, precision medicine, omics, cancer, medical imaging

## Abstract

Cancer is a major medical problem worldwide. Due to its high heterogeneity, the use of the same drugs or surgical methods in patients with the same tumor may have different curative effects, leading to the need for more accurate treatment methods for tumors and personalized treatments for patients. The precise treatment of tumors is essential, which renders obtaining an in-depth understanding of the changes that tumors undergo urgent, including changes in their genes, proteins and cancer cell phenotypes, in order to develop targeted treatment strategies for patients. Artificial intelligence (AI) based on big data can extract the hidden patterns, important information, and corresponding knowledge behind the enormous amount of data. For example, the ML and deep learning of subsets of AI can be used to mine the deep-level information in genomics, transcriptomics, proteomics, radiomics, digital pathological images, and other data, which can make clinicians synthetically and comprehensively understand tumors. In addition, AI can find new biomarkers from data to assist tumor screening, detection, diagnosis, treatment and prognosis prediction, so as to providing the best treatment for individual patients and improving their clinical outcomes.

## Graphical Abstract

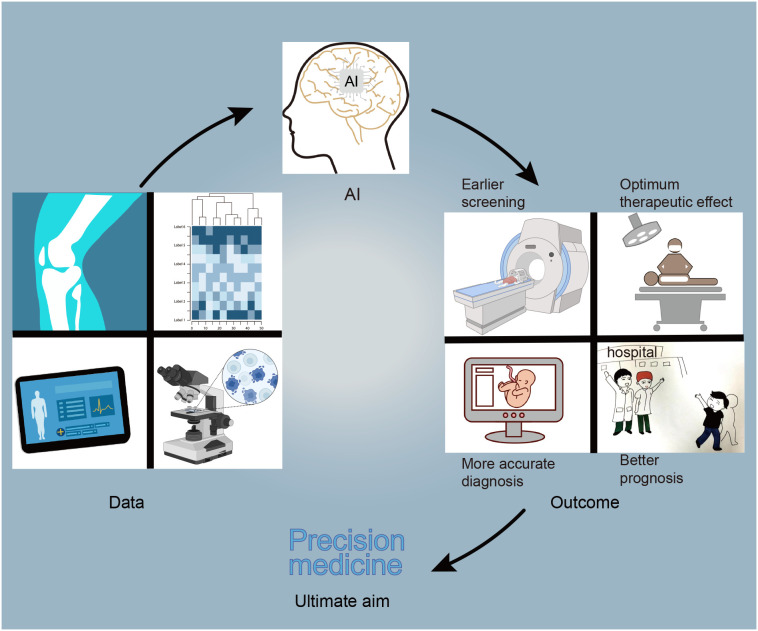

## Introduction

Cancer is a severe threat to human health with a high mortality and a rising incidence rate (1). Several types of cancer can be cured if they are diagnosed and treated early. However, the treatment of cancer is not ideal at present. Cancer mortality rates remain high and continue to rise, including for prostate, colorectal, and cervical cancer (2). These tumors lack effective screening and treatment methods, resulting in patients not getting timely and effective treatment. Secondly, the heterogeneity of tumors is high, which can create great challenges in their treatment (3). Therefore, new diagnostic and treatment methods that are tailored to individual patients are needed. Precision medicine (PM) is a promising approach that takes individual genetics, environment and lifestyle into account and concentrates on clarifying, diagnosing and treating diseases to create a customized treatment plan for patients through obtaining multi-omics or multi-mode information from individuals (4). Furthermore, artificial intelligence (AI) uses computers or machines to carry out tasks by mimicking or emulating human intelligence, which mainly includes machine learning (ML) and deep learning (DL) (5). AI can process an enormous amount of information to promote the brand-new discovery of PM. AI has shown extraordinary potential in processing, mining and analyzing data and can use the data to develop different models to help achieve PM.

**Figure 1:**
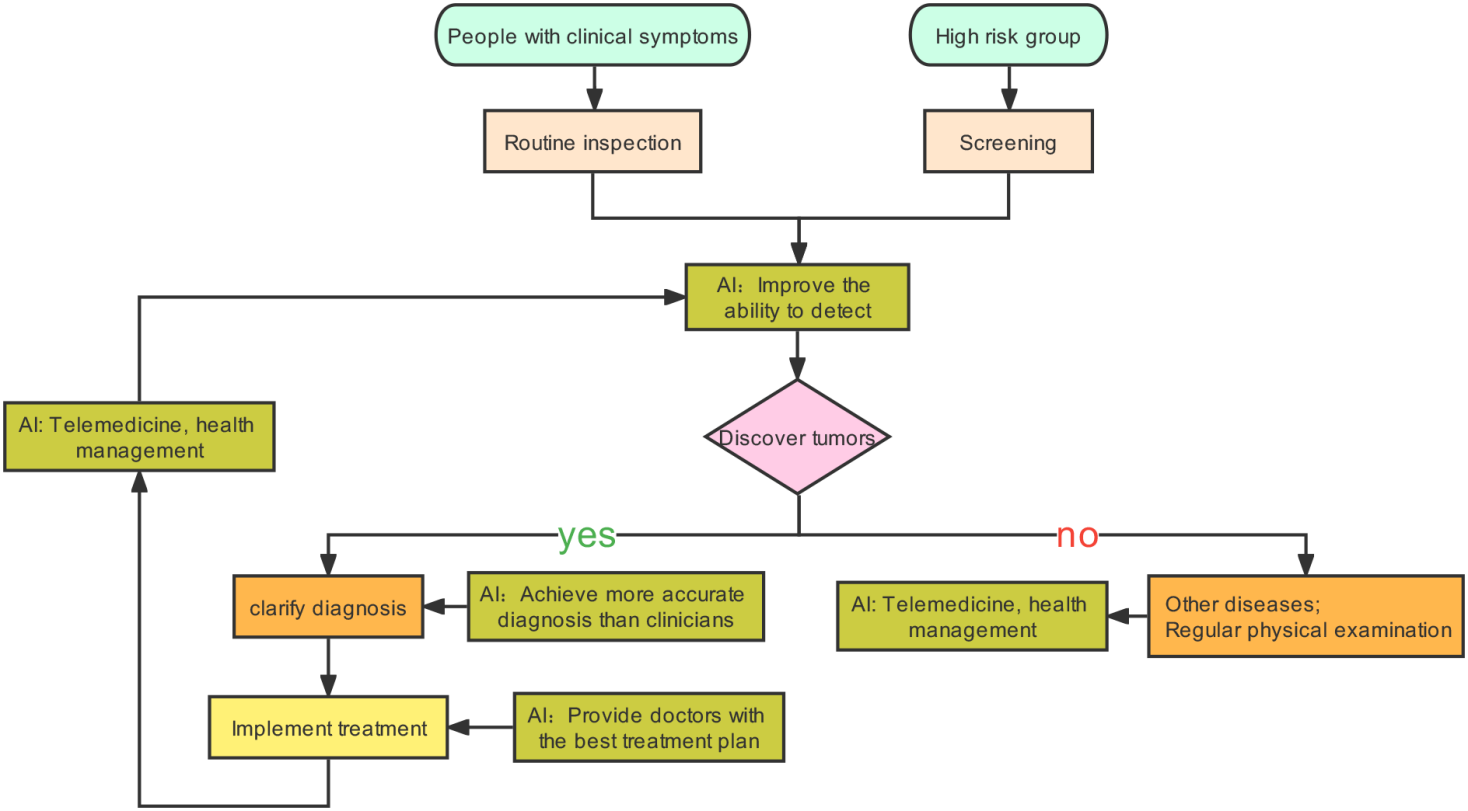
Possible changes caused by AI injection into clinical practice.

Tumors are generally caught sight of in the following two situations: one is the screening of high-risk groups (6). The other one is the discovery of tumors with clinical manifestations. After the cancer is detected, patients will receive further examinations, such as physical examination, imaging, pathology, and serum tumor markers (6). Based on these results, tumors will be accurately diagnosed, staged, and classified to help the patients benefit from precision treatment. AI can play a part in tumor prevention, screening, diagnosis, treatment, and prognosis prediction (7–10). After AI is injected into the clinical process, it will improve the detection rate of lesions and make the screening method more effective. Secondly, AI can promote the level of diagnosis by helping doctors distinguish between true and false disease progression (7). Finally, AI can calculate the advantages and disadvantages of each treatment scheme and provide the best treatment for patients. In addition, a framework diagram (**Figure 1**) is added to this article, which shows a series of processes from the discovery of tumor patients to the end of their diagnosis, treatment, and the changes that AI can bring.

**Figure 2:**
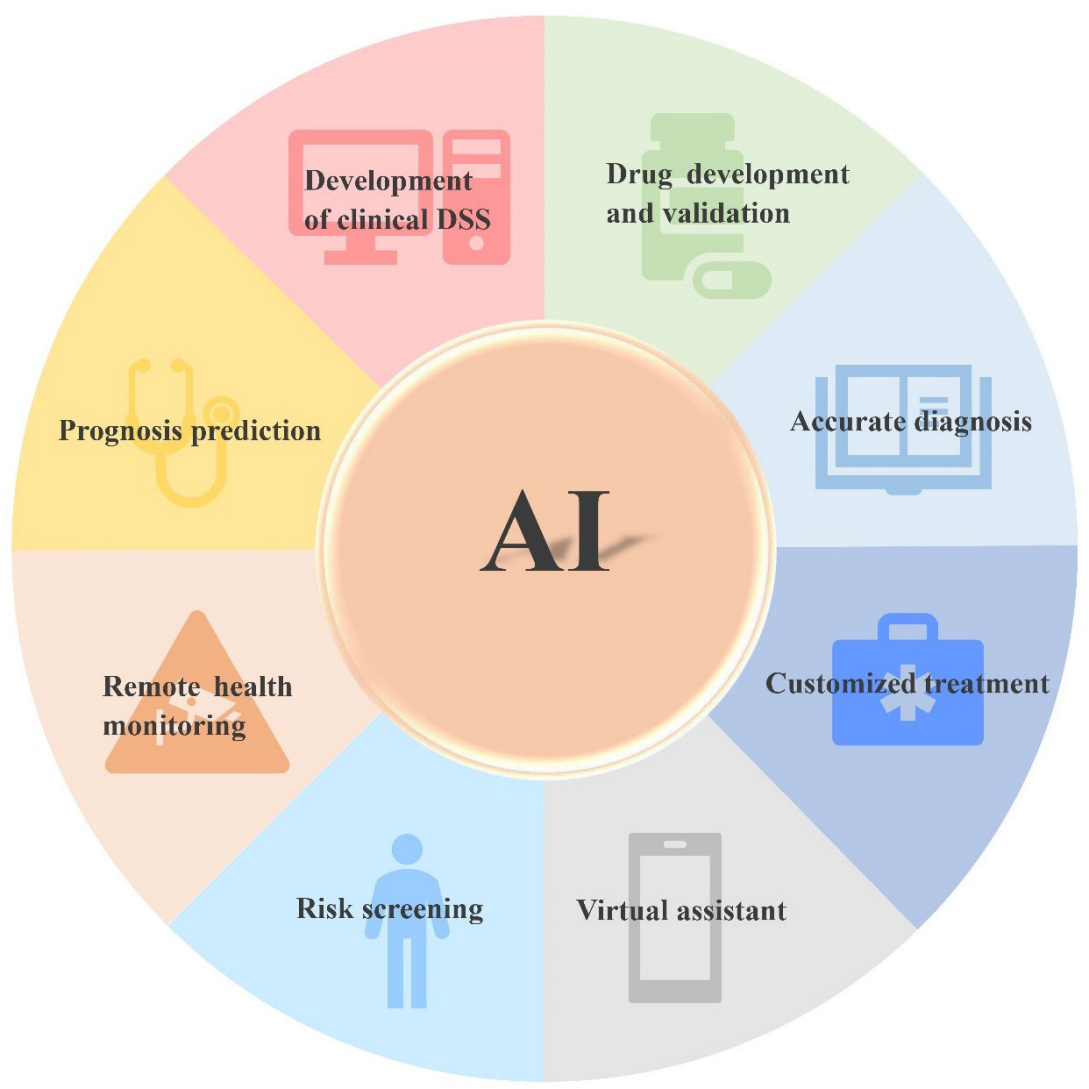
Application prospect of AI in tumor.

With the development of next-generation sequencing (NGS) technology, omics data, such as genomics, proteomics and transcriptomics, have been accumulated (11). Meanwhile, the massive growth and wide availability of patients’ clinical data such as electronic medical records, clinical trial data, and medical images have led to the era of “big data” (12). The best analysis method is data analysis based on AI, since ML and DL can extract the hidden patterns, important information, and corresponding knowledge behind the data. Based on extracted data, information about the disease is obtained to help clinical analysis. For example, ML and DL can be used to analyze omics data to establish models, generate biomarkers related to diagnosis, classification, and prognosis, provide molecular changes such as DNA, RNA and protein, predict drug efficacy and therapeutic response, and develop targeted drugs (13). Furthermore, as compared with single-omics, multi-omics provides an opportunity to understand the information flow behind a disease (14). Multi-omics integration is crucial to the comprehensive understanding of complex biological processes. Combined with the new longitudinal experimental design, multi-omics can clarify the dynamic relationship between all layers of omics, distinguish the key roles or interactions in system exploitation or complicated phenotypes, clarify the causal relationship and functional mechanisms of complicated diseases, and promote the discovery of PM (15, 16). Quantitative image analysis is a suitable candidate for PM and can assist PM for cancer. ML and DL have been used for quantitatively extracting image features to establish models for diagnosis, monitoring, and predicting recurrence and metastasis, biomarkers and prognosis (17–21). AI can integrate the above data for comprehensive analysis of tumors for the development of a clinical decision support system (DSS) (22). With the continuous improvement of AI algorithms and the improvement of computer software and hardware, AI will mature and will be used more extensively in the medical field (**Figure 2**) in the future. Therefore, PM for tumors will great evolve.

In the present review, we first introduced the application of AI in omics, and then in pathology and medical imaging, and expanded on how these applications assist PM. Finally, we described the challenges and future directions of AI assisted PM for tumors.

## AI-based big data assists PM for cancer

Big data technology mainly includes data analysis, mining, and sharing. It may play a revolutionary role in cancer diagnosis, treatment, prevention, and prognosis, but transforming data into available information to benefit patients is almost at a standstill (23, 24). A major reason for this is that data analysis significantly lags behind data generation (24). The reforms caused by “big data” have affected nearly all aspects of tumor research. For example, the technology can analyze data generated by NGS to discover commonly mutated genes, abnormal gene expression, and biomarkers in tumors for accurate diagnosis and prognosis prediction or to determine the cause of disease and develop targeted drugs for treatment (23, 24). The technology can analyze features that humans can and cannot see in medical images, and mine and filter these features to determine information related to diagnosis, treatment, and prognosis (25, 26). In addition, the technology can analyze patients’ demographic and clinical data, as well as outcome information to predict the factors affecting the prognosis of cancer patients (27). In addition, AI is used to analyze, mine and process tumor-related data, build a health care provider platform based on a significant quantity of tumor-related data, efficiently solve the problem of difficult medical treatment for patients and reduce the waste of unnecessary medical resources (28). Big data reanalysis has been not been sufficiently taken advantage of so far, but we cannot ignore its potential. It can analyze the data in an existing database and provide new insights. For example, Borziak et al. discovered the dedifferentiation markers of liver cancer by using data from existing databases (29). The current big data technology is mainly used in certain fields, such as omics, pathological imaging and medical imaging. However, it does not combine data from multiple fields for data analysis, mining, and sharing, which leads to data not being comprehensively utilized and not meeting clinicians’ and patients’ needs. The challenges in the diagnosis, treatment and monitoring of cancer can be overcome by integrating omics and non-omics data. AI can play an important role in analyzing high-dimensional data-sets with complexity and heterogeneity, especially in multi-omics, intergroup methods and data integration, thus setting forth the cancer molecular mechanism, and identifying new dynamic diagnostic and prognostic biomarkers to provide accurate cancer care (30).

There are certain problems with the current data, such as poor data quality, unstructured databases, inadequate analytics, and lack of delivery (23, 31). Therefore, there is a need for a more authoritative and reliable prospective database. In addition, a longitudinal database is also needed to understand the cancer dynamics of patients in the whole study care continuum (23). Establishing a patient-centered collection of various data-sets will be crucial in the future (32). On this basis, AI-based big data analysis may automatically generate patient diagnosis, personalized treatment plans, and key information for prognostic prediction, thereby helping clinicians provide the best treatment for their patients.

## AI assists tumor PM in omics

**Figure 3:**
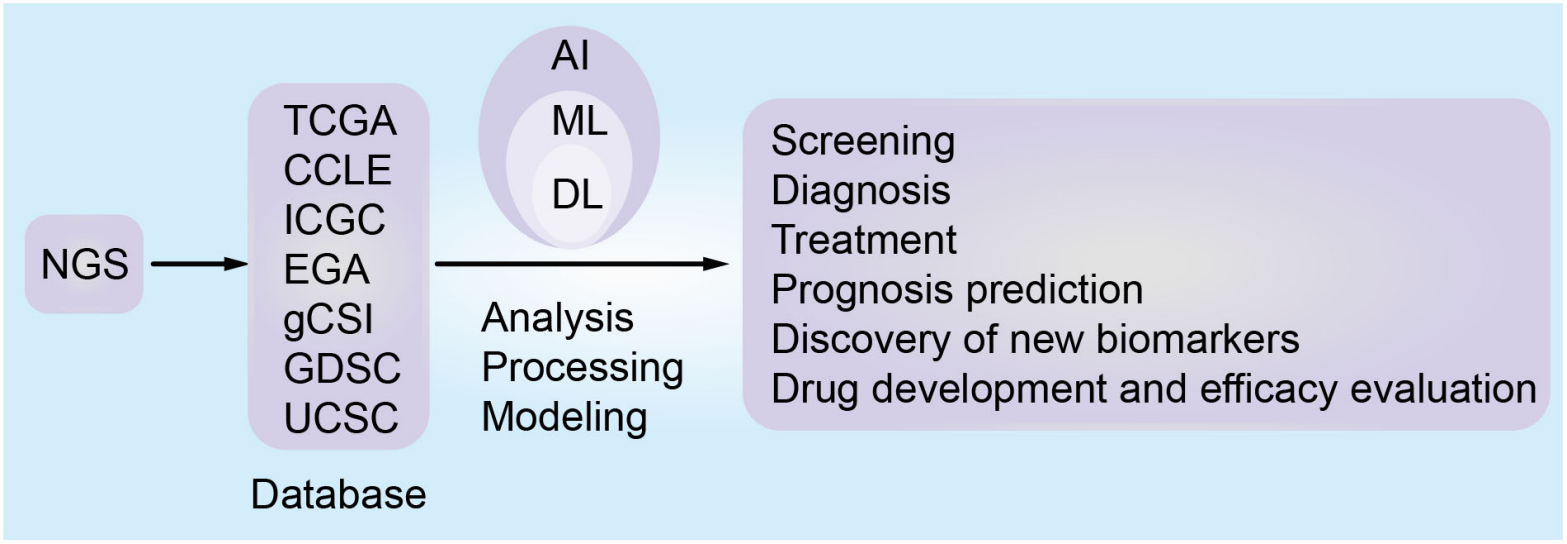
The role of artificial intelligence based on omics database in tumor precision medicine.

A large amount of data resources (**Table 1**) generated by NGS can provide key information about tumors. Combining the information with AI will help clarify the etiology and pathogenesis of tumors, and assist the accurate diagnosis, risk stratification, and disease subtype analysis (30, 33). Moreover, AI can identify new therapeutic targets, evaluate the sensitivity and resistance of anticancer drugs, develop new targeted drugs, improve cancer immunotherapy, monitor the recurrence and evolution of the tumor, discover new biomarkers, and predict the prognosis and survival analysis of tumor patients (**Figure 3**) (34–40). In a few words, AI enables PM for cancer patients, bridging the distance between omics and the clinic. Since NGS produces high-dimensional and complex data, NGS methods for cancer diagnosis usually need higher-dimensional and deeper-seated data coverage to enhance the possibility of detecting a small number of tumor cell mutations and improve the sensitivity and accuracy of AI algorithms (41).

**Table 1:** Comprehensive omics database resources for building AI models.

Name	Main features	Web link
CGHub	Overall data repository; enormous data	https://cghub.ucsc.edu/
TCGA	Comprehensive database; enormous data	https://www.cancer.gov/about-nci/organization/ccg/research/structural-genomics/tcga
CCLE	Comprehensive database; enormous data	https://sites.broadinstitute.org/ccle
EGA	Overall data repository; enormous data	https://ega-archive.org/
ICGC	Comprehensive genomics data	https://dcc.icgc.org/
DepMap	High data quality; visualization	https://depmap.org/portal/
SomamiR	Correlation between cancer somatic mutation and miRNA	https://compbio.uthsc.edu/SomamiR/
COSMIC	largest and most comprehensive somatic mutation database; regularly-updated	https://cancer.sanger.ac.uk/cosmic
MethyCancer	integrated data of DNA methylation, cancer-related gene, mutation and cancer information	http://methycancer.psych.ac.cn/
CTRP	connecting sensitivity to cancer feature	https://portals.broadinstitute.org/ctrp/
gCSI	Large amount of transcriptomics data	https://pharmacodb.pmgenomics.ca/datasets/4
GDSC	Drug response data; genomics markers of drug sensitivity; update irregularly	https://www.cancerrxgene.org/
NCI60	Large amount of drug data and genomics data	https://discover.nci.nih.gov/cellminer/loadDownload.do https://dtp.cancer.gov/databases_tools/bulk_data.htm
canSAR	Comprehensive database; discovery drug	https://cansarblack.icr.ac.uk/
cBioPortal	Large amount of available data	https://www.cbioportal.org/datasets
UCSC	Synthetical genomics information	https://genome.ucsc.edu/
dbNSFP	Predictive data	http://bib.oxfordjournals.org/
NONCODE	database dedicated to non-coding RNAs	http://www.noncode.org/
CSD	The positive and negative training sets	http://bib.oxfordjournals.org/
TCIA	A great quantity of medical related image data sets	https://www.tcia.at/home
MSKCC	Cancer mutation databases	http://www.cbioportal.org/
ARCHS4	comprehensive processed mRNA expression data	https://maayanlab.cloud/archs4/

## AI assists tumor PM in genomics

In recent years, genomics, which relies on nucleotide sequences for data analysis, has become more closely combined with clinical practice (30). The significant accumulation of data has improved the understanding of cancer vulnerability and has enabled us to increasingly anticipate noticeable treatment effects for tumor patients (42). The use of spatial and single-cell genomics may reconstruct the process of tumorigenesis to facilitate a more comprehensive understanding of tumor, decipher the unclear pathogenesis of human beings and develop targeted drugs based on this mechanism (43–47). The combination of ML and genomics data can assist the diagnosis of cancer subtypes, discovering new markers and drug targets, and understanding cancer-driving genes better, which contributes to providing customized treatment for patients (48). For example, Wang et al. developed a compounded deep network model that can diagnose lung cancer subtypes by mixing image-genomics data and can help biomedical professionals determine the potential therapeutic targets by the attention weights of the model (49). In addition, Vanderbilt et al. developed and validated a brand-new approach to identify DNA viruses from corresponding normal or tumor NGS specimens and inquire about virus-tumor type relevance without carrying out extra sequencing. Data on these viruses can provide information for the diagnosis and care of tumor patients. Their study illustrated the function of DNA viruses in the tumor (50). Sudhakar et al. used cancer genomics data and built a pan-cancer model to forecast and identify new driver genes (51). The identification of driving genes can help understand the carcinogenic mechanism and the design of treatment strategies, which has an important biological and clinical significance (52).

## AI assists PM for tumors in transcriptomics

Transcriptomics is a powerful means to evaluate all transcripts produced during metabolism (30). Transcriptomics have expanded our knowledge of cancer occurrence and development, tumor microenvironment, and immune-oncology, and can directly determine gene expression levels and analyze the activation of related molecular pathways (53, 54). Transcriptomics is a bridge between genomics and proteomics, mainly involving quantitative reverse-transcription-polymerase chain reaction, microarrays and NGS (RNA-sequencing) (55). Since RNA-sequencing has a higher accuracy in measuring gene expression, it is considered the gold standard for high-throughput gene expression screening (55, 56). Through data mining or more complex mathematical approaches using ML or DL, the features are extracted to facilitate tumor screening and early diagnosis, discover new or previously unknown cancer biomarkers and potential therapeutic targets, as well as drug prioritization, and predict cancer drug sensitivity and prognosis (53–55, 57–62). For example, Warnat-Herresthal et al. found that ML-based transcriptomics can assist in the diagnosis of acute myeloid leukemia (63). Moreover, Ben Azzouz et al. used an ML approach based on transcriptomics data to calculate triple-negative breast cancer subtypes, in order to overcome the barrier of heterogeneity in the treatment of the disease (64). Finally, some ML-based transcriptomics have also been used in the development of prognostic biomarkers for prostate cancer (65), the diagnosis of colorectal cancer (66), and the prediction of immune response (67).

## AI assists tumor PM in proteomics

Proteomics can provide comprehensive and quantitative information about proteins in tissues, blood and cell samples (68). Protein expression profiles generated by proteomics and ML-based profile analysis can identify more specific and sensitive protein biomarkers than other single-omics. These biomarkers can diagnose cancer, predict prognosis (69), reveal critical signaling pathways behind disease mechanisms (70, 71), determine new therapeutic targets, evaluate drug therapy efficacy and toxicity (72), and predict therapeutic responses, recurrence, and metastasis (73, 74). Recently, Henry et al. proposed a method of drug ranking using ML to predict drug response using proteomics data, and prioritize drugs in order to identify the most suitable drug for each patient (75). In addition, Federica et al. built a clearer and more transparent DSS to assist in diagnosing high-grade serous ovarian cancer (76). Therefore, AI-based proteomics may play an important role in the accurate diagnosis and treatment of tumors in the future.

Besides the widely used omics data mentioned above, other omics data (metabolomics, immunomics and microbiome data) are also used (77). For instance, disposing of metabolomics data by AI can assist the diagnosis (78, 79), the of treatment response evaluation (80–82), discovery of new biomarkers (83, 84), and determination of patient tolerance (85) and cancer status (invasive or non-invasive) (86). Moreover, the AI model based on immunomics data can forecast the emergency immune characteristics of tumor patients (87).

## AI assists tumor PM in multi-omics

Although the current single-omics data can be used for diagnosis, treatment and prediction, they cannot thoroughly and systematically reflect the molecular changes of a tumor (88). Therefore, it is necessary to integrate multi-omics data to comprehensively understand the tumor information and its dynamic development process to screen and accurately diagnose patients, develop tailored treatment strategies, predict prognosis, and monitor recurrence and metastasis (89–92). Some approaches and algorithms of using AI to analyze multi-omics data comprehensively include clustering, factorization, feature transformation, networks-based means and feature extraction (89). These approaches can be used for stratifying medicine, discovering biomarkers (93), pathway analysis, and drug reuse or discovery (89, 94) (**Table 2**). For example, Ma et al. introduced a new approach that can analyze multi-omics information and related knowledge to reveal the complex relationship between molecular features and clinical characteristics (114). In addition, Wang et al. developed a molecular algorithm for early cancer detection, which is used to confirm malignant cellular tumors according to the spectrum of changes in single-cell copy numbers based on doubtful cells in humoral, resulting in a well-defined cancer diagnosis (115). Furthermore, Olivier B et al. have developed an integrated framework of DL and ML, which can use multi-omics data to accurately predict survival and prognosis (95). Furthermore, except for the above commonly used omics, studies have also focused on linking radiomics with genomics and transcriptomics for accurate diagnosis (116). A multi-task DL framework called OmiEmbed, which can analyze and process several kinds of omics data and simultaneously handle multiple tasks has recently emerged. This disruptive technological breakthrough will significantly promote the development of PM (117). The application of AI to integrate multi-omics data is shown in **Table 2**.

**Table 2:** Application of AI in the Integration of Multi-omics.

Clinical application	Data	Model/Algorithm	Performance	References
cancer prognosis and survival prediction	RNA-Seq, Methylation, and miRNA	semi-supervised flexible hybrid machine-learning framework	Not applicable	Poirion, O.B., et al. (95)
breast cancer subtype identification	mRNA expression, miRNA expression and DNA methylation	deep learning fusion clustering framework	0.664	Shuangshuang, L., et al. (91)
cancer susceptibility prediction	copy number variations, miRNA expression, and gene expression	multimodal convolutional autoencoder model	0.9625	Karim, M.R., et al. (96)
identifying Neuroblastoma subtypes	gene expression, copy number alterations, Sequencing Quality Control project	deep learning	0.74	Zhang, L., et al. (97)
predict the survival of patients with lung cancer	TCGA	unsupervised learning	0.99	Takahashi, S., et al. (90)
survival stratification of gastric cancer	transcriptomics and epigenomics	bidirectional deep neural networks	0.76	Xu, J.M., et al. (98)
pan-cancer metastasis prediction	RNA-Seq, microRNA sequencing, and DNA methylation	deep learning	0.8885	Albaradei, S., et al. (92)
ovarian cancer subtypes identification	mRNA-seq, miRNA-seq, copy number variation, and the clinical information	deep learning	0.583	Guo, L. Y., et al. (99)
drug repurposing	copy number alteration, DNA methylation, gene expression, pharmacological characteristics for cancer cell lines	deep learning	0.84	Wang, Y., et al. (94)
predicting lung adenocarcinoma prognostication	mRNA, miRNA, DNA methylation and copy number variations	deep learning	0.65	Lee, T.-Y., et al. (100)
Diagnostic Classification of Lung Cancer	mRNA expression, miRNA-seq data, and DNA methylation data	deep transfer Learning	0.824	Zhu, R., et al. (101)
predicting effective therapeutic agents for breast cancer	copy number variations, miRNA, mutation, RNA, protein expression and methylation	deep learning	0.94	Khan, D. and S. Shedole (102)
predicting survival prognosis for glioma patients	transcription profile, miRNA expression, somatic mutations, copy number variation, DNA methylation, and protein expression	deep learning	0.990	Pan, X., et al. (103)
Diagnostic classification of cancers	mRNA expression, miRNA-seq, DNA methylation data and clinical information	XGBoost	0.595-0.872	Ma, B., et al. (104)
identify tumor molecular subtypes	copy number, mRNA, miRNA, DNA methylation and other omics data	consensus clustering and the Gaussian Mixture model	Not applicable	Yang, H., et al. (105)
predicting outcome for patients with hepatocellular carcinoma	DNA methylation and mRNA expression data	unsupervised machine-learning	Not applicable	Huang, G. J., et al. (106)
predicting the Gleason score levels of prostate cancer and the tumor stage in breast cancer	gene expression, DNA methylation, and copy number alteration	gene similarity network based on uniform manifold approximation and projection and convolutional neural networks	0.99	ElKarami, B., et al. (107)
patient classification, tumor grade classification, cancer subtype classification	mRNA expression, DNA methylation, and microRNA expression data	Multi-Omics Graph cOnvolutional NETworks	Not applicable	Wang, T. X., et al. (108)
cancer prognosis prediction	mRNA, miRNA, DNA methylation, and copy number variation	denoising Autoencoder	Not applicable	Chai, H., et al. (109)
cancer subtype classification	gene expression, miRNA expression and DNA methylation data	hierarchical integration deep flexible neural forest framework	0.885	Xu, J., et al. (110)
Prediction of prognosis of cancer	single nucleotide polymorphism, copy number variant, gene expression, and DNA methylation data	deep learning	0.67-0.88	Park, C., et al. (111)
tumor Stratification	deoxyribonucleic acid methylation, messenger ribonucleic acid expression data, and protein–protein interactions	Network Embedding; supervised learning; unsupervised clustering algorithm	0.91	Li, F., et al. (112)
discovery of cancer subtypes	mRNA expression, miRNA expression, DNA methylation, and copy number alterations	end-to-end variational deep learning-based clustering method; Variational Bayes	Not applicable	Rong, Z., et al. (113)

## AI in pathology assists the accurate diagnosis of tumors

Pathological analysis is considered the gold standard of the clinical diagnosis of tumors (118). However, the current shortage of clinical pathologists and their reliance on subjective consciousness for diagnosis leads to low repeatability and unequal diagnostic levels of clinical pathologists, which is not helpful for clinicians’ decision-making with regards to treatment (119). Computational pathology has seen significant developments from the use of improved AI algorithms and computing power. With the use of image analysis of digital pathology, ML and DL, AI has been used to evaluate whole slide imaging (WSI) and produce computer-aided diagnosis systems (CADs), as well as aid cancer prognosis prediction (120–124). At present, the diagnostic ability of the AI-based diagnostic model can be comparable to or even surpass that of experts (125). In combination with human experts, the precision of diagnosis can be even better. It also has the advantages of being less time-consuming, and having a high efficiency and repeatability. Therefore, an increasing number of AI models are being developed to assist clinical pathologists and reduce their workload (120). For example, Ho et al. proposed incorporating AI models into the pathological workflow as the first reader, the second reader, triage, and pre-screening (120, 126, 127).

**Figure 4:**
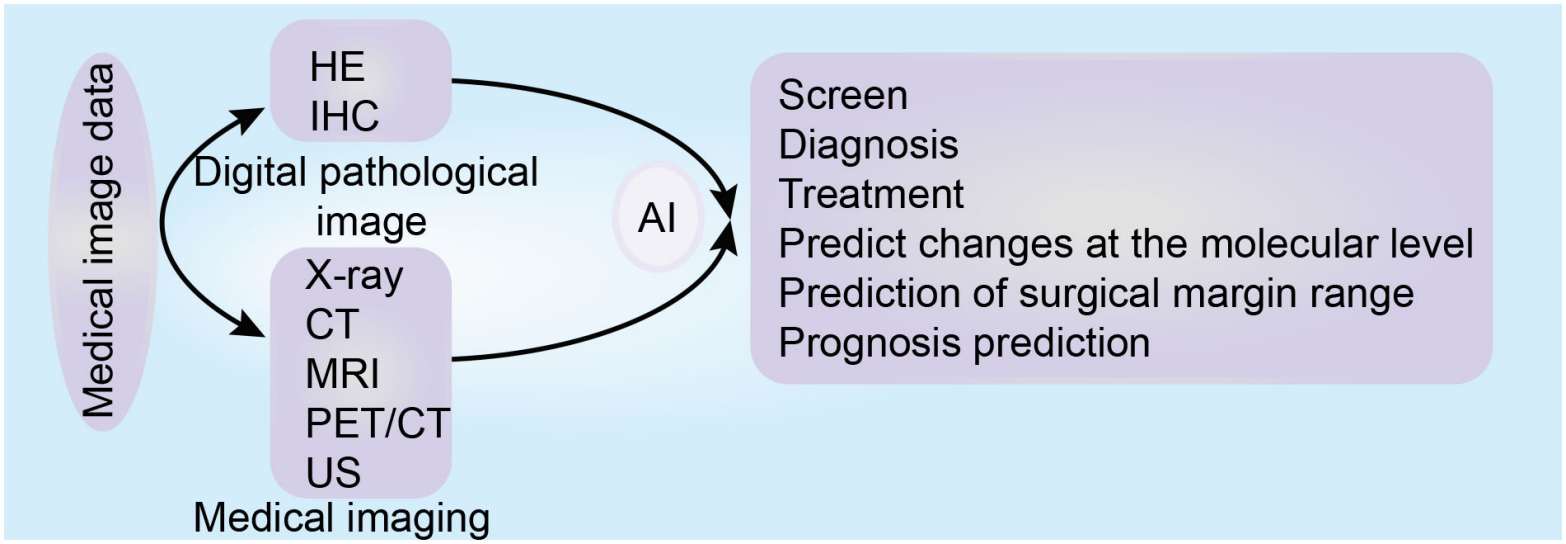
The role of artificial intelligence based on medical images (digital pathological image and medical imaging) in tumor precision medicine.

Aiding diagnosis through ML and DL mainly includes three steps: The first step includes data preprocessing, such as image sharpening, masking and smoothing, image graying and color normalization, data standardization, and data annotation. The second step includes the division of nucleus/tissue. During the third step, models are established for training and verification, diagnosis and prediction (128, 129). For example, the computer-aided diagnosis and prognosis prediction model of WSI based on hematoxylin and eosin staining, can screen, classify and grade tumors (130), and identify micro-metastasis in lymph nodes (25), and microsatellite instability (128). It can also predict the changes at the molecular level (131), the risk of metastasis and recurrence after surgical resection (132) and disease-specific survival (133). Moreover, Armin et al. used a DL model based on digital images of immunohistochemistry to calculate the risk of mortality (134). The role of AI in the digital pathological image is summarized in **Figure 4**.

Traditional ML methods analyze pathological tissue by manually extracting mainly morphological, textural (135) and spatial features (118). It is easier to understand and explain than DL, and its training sample size is small, especially suitable for the analysis of rare tumor subtypes with a limited sample size. However, manually extracted features have the following limitations (118): They are extracted in an unsupervised way and have nothing to do with the subsequent WSI analysis tasks (136). Only the surface features of the input image can be learned, which is not enough to show the complex features of WSI. It is exceptionally arduous to process multiple WSI images at the same time and the processing speed is slow (137). Compared with ML, DL can automatically extract the features in the image for analysis and can also efficiently process a considerable amount of data (129). The DL model has good scalability (138), but it is easy to overfit, resulting in the low generalization ability of the model. Furthermore, it is characterized by low interpretability and cannot be trusted by clinical pathologists (139). Recently, diagnostic models combining various methods of traditional ML, DL have been developed to integrate their advantages for accurate diagnosis and prediction (129). For example, Sengupta et al. have proposed a novel deep hybrid learning model based on nuclear morphology for accurately diagnosing ovarian cancer (129). It can be safely assumed that in the near future more high-performance prediction models will be developed and enter the clinic to assist clinical pathologists in accurate diagnosis and prognosis prediction, consequently providing accurate and personalized medical care for patients.

## AI assists PM for tumors in medical imaging

Imaging is one of the indispensable tools for screening, diagnosis, treatment and follow-ups for several types of tumors. At present, the performance of imaging examination equipment such as thin-layer computed tomography (CT) and multi-parameter magnetic resonance imaging (MRI), is continuously improved, detecting more subtle lesions and producing increasingly complex data. However, this requires more time and effort from radiologists to make a diagnosis, increasing their workload. AI-based data analysis can effectively process huge amounts of data, with the CADs model based on medical images exhibiting high precision and standard (140–142). Introducing AI into clinical practice will help radiologists make diagnostic decisions quickly, accurately, and efficiently, will help focus their energy on advanced decision-making, and promoting accurate medical treatment and personalized treatment for tumors (141, 143). The role of AI in medical imaging is summarized in **Figure 4**.

## AI assists radiologists in accurately diagnosing tumors

AI has three main tasks in tumor imaging: Detecting, characterizing and monitoring tumors (144). Detection refers to the location of the region of interest in the image. Characterization includes tumor diagnosis, and staging. Monitoring refers to the monitoring of the changes in tumors with time (144). The process of ML-assisted tumor detection and diagnosis is as follows: Image data acquisition, image preprocessing, segmentation of regions of interest, feature selection, establishing the model and carrying out training, verification and testing (145). Among them, feature selection is the most important step, since it is most related to the model’s performance (145). Moreover, DL can automatically extract the feature from the image. Therefore, recent research has increasingly focused on the DL to build “fusion” models for the diagnosis of tumor lesions, including classification, grading and staging, which have been proven to be effective (146–148). For example, Chougrad et al. have built CADs based on deep convolutional neural networks (CNN) to aid radiologists in categorizing breast X-ray masses (149). Moreover, Misra et al. have developed a highly robust DL model for categorizing benign and malignant neoplastic lesions of the breast. The model can improve the accuracy of breast cancer classification by correcting patients whose traditional methods are misclassified (150). Overall, these models can make radiologists more effective in detecting and diagnosing tumors faster, and will likely be popularized and applied to clinical medical treatment soon.

## AI assists PM for tumors diagnosed *via* medical imaging

The choice of treatment depends on the outcome of the diagnosis. For example, if the detected lesion is benign, it can reduce unnecessary surgical resection and other treatments, and provide more targeted medical management for patients. In addition, the use of radiation imaging, a non-invasive diagnostic method, can protect patients from the discomfort caused by biopsy and avoid the risk of implant metastasis in pathological biopsy (151). Moreover, preoperative evaluation of tumor grading prediction using radiology can help select more appropriate treatment options for patients and avoid unnecessary surgery, thereby reducing the patient’s medical burden and avoiding excessive medical treatment (152, 153).

In addition to assisting in accurate diagnosis, AI can also play a significant role in prognosis prediction and treatment of patients. It can predict patient viability based on imaging features and determine the level of treatment needed to achieve optimal survival. The prediction of recurrence, metastasis, surgical margins and therapeutic responses can be used to formulate an optimal therapeutic strategy for individual patients (21, 26).

Accurately identifying and evaluating lesion before an operation can help create appropriate treatment plans for patients and avoid unnecessary treatment measures such as surgery, postoperative radiotherapy, and chemotherapy, which is beneficial to both patients and doctors. For instance, Zhao et al. built a neoplasm grade forecast model of pre-operative G1/2 assessment of nonfunctional pancreatic neuroendocrine tumors by using radiomics to analyze the multi-slice helical CT images (152). In addition, Xie et al. used a CT-based radiomics ML method to distinguish pancreatic mucinous cystic neoplasm from atypical serous cystadenomas prior to surgery (154). The classification and types of tumors are different, and their treatment methods are inconsistent. For example, according to the model established by Zhao and Xie, if the preoperative prediction result is a high-risk or high-grade tumor, it is necessary to strengthen the follow-up treatment, such as postoperative neoadjuvant chemotherapy or radiotherapy (152, 154). Depending on whether the lymph node is metastatic or not, clinicians will choose different treatment options for patients. Therefore, the detection of lymph node metastasis is extremely important. For example, Song et al. established and verified a radiomics nomogram based on dynamic contrast-enhanced MRI, which can predict metastasis of axillary lymph nodes in mastocarcinoma (155). Similarly, Eresen et al. used the radiomics-derived model established by ML to detect metastatic lymph nodes in colorectal cancer patients (156). Predicting preoperative tumor markers and imaging biomarkers can lead to better clinical decision-making and help provide the best treatment for patients. For example, Guo et al. developed LR and LR-SVMSMOTE models based on CT radiomics to predict thyroid cartilage invasion in certain cancers types, such as hypopharyngeal squamous cell carcinoma and laryngeal carcinoma (157). Similarly, Akbari et al. combined the advanced mode analysis and ML method of multi-parameter MRI to provide the prediction space map of tumor invasion and early recurrence possibility to provide more targeted surgical and radiotherapy strategies for tumor patients, aiming to maximize the treatment effect while maintaining neurological function (158).

The DL model based on one of the subsets of AI can assist radiotherapy or oncology doctors in accurately outlining tumor targets, reducing the time doctors take to manually segment images as well as reducing the variation between observers (159–163). The model can predict and verify the therapeutic dose, and allows for the dose prescription to be changed in time to reduce the impact on the surrounding normal tissue, prevent unnecessary radiation, and reduce the occurrence of adverse reactions (164, 165). The model can evaluate the efficacy of radiotherapy and chemotherapy, as well as the therapeutic response, so as to achieve better-personalized prescriptions for patients (166–168). For example, Ermiş et al. have used DL to depict fully automatic brain resection cavity delineation in patients with glioblastoma (169). Likewise, Zhou et al. have developed and tested a three-dimensional DL model capable of predicting the dosage distribution of three-dimensional volume units to carry out intensity-modulated radiation therapy (170). Establishing a dose distribution model prior to treatment helps adjust the dose distribution in advance and reduce the probability of complications from radiotherapy. ML and DL can also assist in post-radiotherapy management, such as distinguishing between the true and false progression of the tumor, radiation necrosis and tumor recurrence, and promoting clinical medical decision-making, thus improving PM (171–174). In addition, the image-based AI model can also assist radiologists in treatment evaluation, including predicting the response of individual cancer patients to chemotherapy or immunotherapy, and monitoring recurrence and metastasis (175–177). Several radiomics-based ML and DL models can predict patient prognosis, such as recrudesce-free and progression-free survival, survival rate, mortality, surgical results, postoperative metastasis and recurrence. According to the prediction results of the model, the corresponding processing is carried out to create a customized treatment scheme for patients and improve the treatment effect and later quality of life. For example, patients with lower overall survival prediction need more intensive treatment. Patients with poor surgical results may want to consider changing the surgical method or choosing non-surgical treatment. Patients with a higher risk of tumor recurrence and metastasis should continue to receive neoadjuvant radiotherapy and chemotherapy (21, 26, 178–180).

## Current challenges and future prospects

Although AI is expected to help improve a series of clinical applications against cancer, it does have some challenges and limitations.

One limitation is the lack of standards and imbalance in the data used to build the model (181, 182). These disordered data will lead to the low robustness of the model and be unfit for constructing a DL model with high generalization and precision (129). For example, medical imaging data is generated under different parameters for different devices (5). Digital pathological images are produced by staining with different dyes. The non-standard operation of pathological specimen collection will also affect the quality of pathological images (122). Irregularities in data collection lead to bias. Omics data are also noisy and heterogeneous (183). These data sets, which are generated by different technologies and standards limit the promotion and generalization of AI models, thus limiting their application in clinical practice. In addition, the sample size of training samples and verification samples used to establish the AI model is small, which can easily cause the overfitting of the model (120). Finally, integrating various types of data, such as genomics, transcriptomics, proteomics, metabolomics, immunomics, electronic health records, clinical medical records, pathology and medical images, will help evaluate the tumor comprehensively and develop the best treatment plan for patients (77, 184, 185). Therefore, it is necessary to establish an extensive comprehensive standardized database. However, many types of data have a multi-scale nature, which makes the mechanical connection between data elusive. The biological knowledge of connecting all these variables in a single model is limited, so many data variables will be omitted from the model development process (186). Recently, some studies have combined dynamic modeling and ML to promote the integration of mathematics and clinical oncology. This method can integrate multiple types of data for personalized prediction to assist PM (187). More in-depth research to promote the combination of mechanical modeling and ML approaches is required in the future, so that mathematical oncology can be introduced into clinics. Building deep fusion models such as multi-modality DL is the primary method to develop AI models that can effectively integrate multimodal data information. However, the current method mainly focuses on representation fusion (feature- and decision-level fusion). The main challenge of this method is that the data is highly dimensional, noisy, heterogeneous, and has a small sample size, and there will be data loss during processing (91, 96, 188, 189). Here are some methods to address these barriers: T-distributed stochastic neighborhood embedding, autoencoder, random forest deep feature selection, a stacked autoencoder, gradient descent method, multi-view factorization autoencoder, co-expression network analysis, and regulation techniques (88, 91, 114, 190–193).

In addition, data from patients are governed by privacy laws (194). The lack of supervision of these data may lead to breaching patient privacy rules; therefore, appropriate intervention and the improvement of laws and regulations are required. Certainly, studies have focused on solving the privacy problem with regards to patient data. Under the same performance, the privacy vulnerability is reduced by vocabulary selection means (194). At the same time, fusing these data should comply with the principles of medical ethics.

Another limitation is that AI algorithms have been regarded as “black boxes” (139), since the process of their output results is unknown and unexplained, which makes clinicians have low trust in AI and a low willingness to introduce it into the clinical workflow (181). Developing a knowledge-embedded DL model for multi-dimensional data fusion is a hopeful means for this problem (195). An increasing number of studies on AI interpretability and transparency are being conducted. The research aims to make AI transparent and interpretable so that its results can be convincing and easy to introduce into the clinical (128). Traditional ML and DL have their advantages and disadvantages, prompting more research on hybrid learning methods. The current research results show that the hybrid learning model exhibits a better performance, better interpretability, higher transparency and more accurate prediction (129, 148). Although AI has shown the ability to surpass people, since it cannot produce 100% correct results, doctors’ participation is still required for the final diagnosis and treatment decisions (183). Future research will focus on improving the interpretability and performance of AI, because it is an important step for AI to realize clinical application (31).

AI model may be necessary to carry out clinical experiments similar to clinical drug trials because when AI models are initially applied to clinical practice, unexpected clinical conditions will inevitably occur. Only through continuous practice can we better find problems and solve them to improve AI models. However, as AI differs from drugs, its clinical trial plan should also be distinguished from drug clinical trials.

AI can be deployed before, during, and after diagnosis, which respectively stands for cancer prevention, screening, diagnosis, and treatment. For example, before diagnosis, AI can be combined with gene detection, endoscopic examination, and other technology to predict the risk of disease occurrence earlier and carry out risk management for patients to reduce the possibility of disease occurrence (196). Augmented or virtual reality can simulate experiences to improve patient compliance (165). During diagnosis, AI can roughly ask patients for relevant information and process it. Secondly, AI can analyze medical image, blood biochemistry, and other clinical overall data to automatically generate a diagnosis report and a variety of feasible as well as optimal treatment methods. Furthermore, after diagnosis, AI can assist clinicians cut down the damage and maximizing the benefits for patients in surgery, radiotherapy, and chemotherapy. The deployment of AI in clinical practice will improve the efficiency of clinicians, reduce the possibility of clinical errors, improve the medical status in areas with low medical levels, and reduce unnecessary procedures, interventions, and medical costs. In a word, patients and doctors will benefit from AI to achieve a win-win situation.

Most people believe that AI cannot replace doctors (7). AI is an assistant in clinical practice, so the final decision must be made by doctors; the responsibility should also be borne by doctors. However, the clinician cannot control AI because it can make self-development and its development process illegible. Therefore, doctors should not be fully responsible for AI errors. Despite that, when using AI, clinicians should not lose their ability to doubt AI to make accurate diagnoses and treatments and develops the doctor-patient relationship in a sound direction.

## Conclusion

AI has shown promising results in certain fields of oncology, including tumor screening, detection, diagnosis, treatment, and prognosis prediction. With the progress of AI, the improvement of computer performance, and the explosive growth of various data, new learning methods, such as the hybrid learning method, will continue to emerge, further improving the overall performance of the model, such as efficient data analysis and accurate prediction. The recent model generated by the ML and DL that can analyze various data sets will also improve the prospects of PM. In conclusion, AI-assisted PM can help detect, diagnose and treat cancer early, as well as assist in the selection of the best treatment scheme, consequently improving the prognosis of patients and improving their treatment results.

## Author contributions

JL collected the related papers and was a significant contributor to writing the manuscript. XL, YG, and SH made figures and tables. PR, WW, WL and LZ revised the article. PR, WW, WL and LZ initiated the study and revised the manuscript. All authors contributed to the article and approved the submitted version.
